# Recombinant Production of the Amino Terminal Cytoplasmic Region of Dengue Virus Non-Structural Protein 4A for Structural Studies

**DOI:** 10.1371/journal.pone.0086482

**Published:** 2014-01-23

**Authors:** Yu-Fu Hung, Olga Valdau, Sven Schünke, Omer Stern, Bernd W. Koenig, Dieter Willbold, Silke Hoffmann

**Affiliations:** 1 Institute of Complex Systems, Structural Biochemistry (ICS-6), Forschungszentrum Jülich, Jülich, Germany; 2 Institut für Physikalische Biologie, Heinrich-Heine-Universität, Düsseldorf, Germany; 3 Department Clinical Microbiology and Immunology, Sackler School of Medicine, Tel Aviv University, Tel Aviv, Israel; University of California, San Francisco, United States of America

## Abstract

**Background:**

Dengue virus (DENV) is a mosquito-transmitted positive single strand RNA virus belonging to the Flaviviridae family. DENV causes dengue fever, currently the world's fastest-spreading tropical disease. Severe forms of the disease like dengue hemorrhagic fever and dengue shock syndrome are life-threatening. There is no specific treatment and no anti-DENV vaccines. Our recent data suggests that the amino terminal cytoplasmic region of the dengue virus non-structural protein 4A (NS4A) comprising amino acid residues 1 to 48 forms an amphipathic helix in the presence of membranes. Its amphipathic character was shown to be essential for viral replication. NMR-based structure-function analysis of the NS4A amino terminal region depends on its milligram-scale production and labeling with NMR active isotopes.

**Methodology/Principal Findings:**

This report describes the optimization of a uniform procedure for the expression and purification of the wild type NS4A(1-48) peptide and a peptide derived from a replication-deficient mutant NS4A(1-48; L6E, M10E) with disrupted amphipathic nature. A codon-optimized, synthetic gene for NS4A(1-48) was expressed as a fusion with a GST-GB1 dual tag in *E. coli*. Tobacco etch virus (TEV) protease mediated cleavage generated NS4A(1-48) peptides without any artificial overhang. Using the described protocol up to 4 milligrams of the wild type or up to 5 milligrams of the mutant peptide were obtained from a one-liter culture. Isotopic labeling of the peptides was achieved and initial NMR spectra were recorded.

**Conclusions/Significance:**

Small molecules targeting amphipathic helices in the related Hepatitis C virus were shown to inhibit viral replication, representing a new class of antiviral drugs. These findings highlight the need for an efficient procedure that provides large quantities of the amphipathic helix containing NS4A peptides. The double tag strategy presented in this manuscript answers these needs yielding amounts that are sufficient for comprehensive biophysical and structural studies, which might reveal new drug targets.

## Introduction

Dengue fever is currently the fastest-spreading tropical disease in the world, with more than 2.5 billion people at risk. Dengue virus (DENV), the causative agent of this disease, is estimated to infect 390 million people across all continents each year [1]. DENV is transmitted by the bite of female mosquitoes. DENV causes flu-like symptoms in most of the infected patients, but severe forms of the disease like dengue hemorrhagic fever and dengue shock syndrome are life-threatening. There is no specific treatment and no anti-DENV vaccines.

DENV is a positive single strand RNA virus of the *Flaviviridae* family. Its genome is translated into a single polyprotein, which is subsequently cleaved into three structural and seven non-structural (NS) proteins. DENV replicates its RNA genome in replication complexes (RCs), which are associated with modified intracellular membranes [2]. While the viral structural proteins compose the mature virion, the NS proteins together with the viral RNA and host factors generate the viral RC. NS4A is an endoplasmic reticulum (ER)-localized, 16 kDa transmembrane protein, which is an essential component of the viral RC. NS4A has been suggested to be involved in inducing host membrane alterations that resemble the virus-induced membrane structures [3]. A membrane remodeling function of NS4A was also reported in other flaviviruses [4], [5].

Recently, we identified a conserved amphipathic helix (AH) in the cytoplasmic, amino terminal region of NS4A (amino acid residues 1–48) that is essential for viral replication [6]. AHs are α helical protein regions in which one face of the helix is hydrophobic while the opposite face is hydrophilic [8]. In contrast to transmembrane domains that span the membrane bilayer, AHs often serve as in-plane membrane anchors [7]. Peptides that are predicted to form amphipathic helices are frequently unstructured in buffer devoid of membranes but adopt a helical conformation upon association with membranes or in a membrane mimicking environment. In addition AHs can contribute to membrane curvature [8]–[10] or mediate protein-protein-interactions. AHs in proteins of several positive strand RNA viruses were shown to be essential for the viral life cycle [11]–[16].

The use of direct acting antivirals is one of the most important new therapeutic approaches for treating infections with hepatitis C virus (HCV), a close relative of DENV. Notably, AHs in HCV NS4B and NS5A were found to be amenable to pharmacological inhibition [11], [17] indicating that AHs could serve as novel antiviral targets. Our recent data emphasizes the significance of the amino terminal AH of NS4A in the DENV life cycle and demarcate it as potential target for the design of novel antiviral therapy [6]. In this report, we describe a novel protocol for recombinant production of a peptide comprising the first 48 amino acids of NS4A, NS4A(1–48), and containing the above mentioned AH. Production of milligram amounts of NS4A(1-48) is a crucial prerequisite for biophysical and in particular NMR experiments on NS4A(1-48). Such studies are urgently needed as a starting point for the rational design of new strategies to inhibit the activity of this NS4A region in the virus life cycle.

It is a common strategy to express short peptides as a fusion with another protein in order to avoid the well-known degradation of short peptides in bacterial cells. However, expression of fusion proteins is complex and does not always follow predictions. Hence, it is necessary to test several fusion strategies in search for a highly efficient protocol that works for a given peptide. Several different proteins have been described in the literature as fusion tags for peptide production [18], [19]. We tested glutathion S-transferase (GST), the immunoglobulin-binding domain of streptococcal protein G (GB1), and yeast ubiquitin as fusion partners of NS4A(1-48) in our quest for an effective production strategy of this peptide in *E. coli*. For DENV NS4A(1-48) a dual fusion tag in combination with a tobacco etch virus (TEV) protease cleavage site revealed the highest peptide yields. Our aim was to establish a general protocol for production and purification of decent amounts of wild type NS4A(1-48) as well as of various mutated forms of the peptide. Such mutants are quite useful in an in-depth structure-function-analysis of proteins.

## Materials and Methods

### Materials

*E. coli* Mach 1 cells obtained from Life Technologies GmbH (Darmstadt, Germany) were used for cloning purposes. *E. coli* BL21-(DE3) (Agilent Technologies, Inc., Santa Clara, CA, USA) or *E. coli* BL21 (GE Healthcare, Freiburg, Germany) strains were used for peptide expression. All enzymes used for cloning were obtained from MBI Fermentas (St. Leon-Rot, Germany) if not stated otherwise. Synthetic oligonucleotides were from BioTez (Berlin, Germany). Plasmid pGEX-4T-2 was obtained from GE Healthcare. The plasmid pTKK19ubi was a kind gift from Toshiyuki Kohno (Mitsubishi Kagaku Institute of Life Sciences (MITILS), Machida, Tokyo, Japan) [20] and pRK793 (plasmid #8827) was obtained from the Addgene plasmid depository (http://addgene.org). PCR purification kit, gel extraction kit (QIAquick), miniprep kit (QIAprep spin) and Ni–NTA agarose were all from Qiagen (Hilden, Germany). Difco LB broth was purchased from BD Biosciences (Heidelberg, Germany). Ampicillin and kanamycin were from AppliChem (Darmstadt, Germany), Isopropyl β-D-1-thiogalactopyranoside (IPTG) was from Boehringer (Mannheim, Germany). [^15^N] ammonium chloride and [^13^C_6_]-glucose were from Euriso-top (Saarbrücken, Germany). Protein standards were obtained from MBI Fermentas and Sigma-Aldrich (Munich, Germany). Glutathion-sepharose 4B was purchased from GE Healthcare. All other chemicals were from Sigma-Aldrich, Roth (Karlsruhe, Germany) or Merck (Darmstadt, Germany) unless stated otherwise.

### Construction of NS4A(1-48) expression vectors

#### NS4A(1-48) plasmid constructs with a single fusion tag

We first cloned NS4A(1-48) into a modified pTKK19ubi vector (see below), which codes for an amino terminal yeast ubiquitin fusion. The peptide bond between ubiquitin and a defined peptide insert in the expressed fusion protein can be cleaved by yeast ubiquitin hydrolase. This strategy enables the production of a NS4A(1-48) peptide without any artificial overhangs. In order to simplify cloning of inserts into pTKK19ubi vector [20], we modified the original pTKK19ubi by introducing silent mutations that generate a unique *Sac*II endonuclease cleavage site at the carboxy-terminal end of the ubiquitin coding sequence. In detail, the original codons for Leu 73, Arg 74 and Gly 75 in the ubiquitin coding sequence of pTKK19ubi are substituted with ctc, cgc, and ggc, respectively, in the new pTKK19ubi/*Sac*II. Plasmid pUbi-NS4A(1–48) was obtained by cloning an optimized sequence coding for amino acids 1 to 48 of NS4A from dengue virus type 2 (GenBank: NP739588) into pTKKubi/*Sac*II. Gene optimization was performed using the GeneOptimizer® software provided by GeneArt® [21]. To construct the plasmid, four complementary synthetic oligonucleotides (sequences 1 to 4, table 1) were annealed, ligated and subjected to a PCR-amplification with two shorter primers (9 and 10, table 1) containing the restriction sites for *Sac*II and *Sal*I. The purified fragment was cut by *Sac*II and *Sal*I and then ligated into dephosphorylated pTKK19Ubi/*Sac*II.

**Table 1:** Primers used for amplification of NS4A (1–48).

Oligo	Sequence (5′-3′)
1	agcctgaccctgaatctgattaccgaaatgggtcgtctgccgacctttatgacccagaaagcacgtgatgcactggataatctgg
2	cagttctgcataccgctgaagccggtggtcgtgcatataatcatgcactgagcgaactgtaag
3	tgctttctgggtcataaaggtcggcagacgacccatttcggtaatcagattcagggtcaggct
4	gatccttacagttcgctcagtgcatgattatatgcacgaccaccggcttcagcggtatgcagaactgccagattatccagtgcatcac
5	ggaggaggatccgaaaacctgtattttcagagcctgaccctgaatctgattacc*Bam*HI
6	ggaggagtcgacctcgagttacagttcgctcagtgc*Xho*I
7	gcgcgaattcagtacaagcttgctctgaacgg*Eco*RI
8	gctagttattgctcagcgg (T7-Terminator-Primer; Novagen #69337-3)
9	ggaggaccgcggcggtagcctgaccctgaatctga*Sac*II
10	ggaggagtcgacctcgagttacagttcgctcagtgc*Sal*I
11	gaaattaccgaagagggtcgtctgccgac
12	attcagggtcaggctctgaaaatacaggtt

Note: The sequences of the restriction sites used for cloning are underlined, the name of the enzyme is given below.

Second, a plasmid for expression of the NS4A peptide as protein fusion with the immunoglobulin-binding domain of streptococcal protein G (GB1) at the amino terminus was obtained by cloning the optimized sequence coding for NS4A(1-48) into the vector pGEV2 [22] using the four synthetic oligonucleotides 1 to 4 (table 1). PCR amplification was performed with different primers (5 and 6) in order to introduce a nucleotide sequence that codes for the first six residues of a tobacco etch virus (TEV) protease cleavage site (ENLYFQ) in front of the NS4A coding sequence. The primers contained *Bam*HI and *Xho*I restrictions sites at their 5′ and 3′ ends, respectively. This fragment was ligated into dephosphorylated pGEV2 to yield pGEV-NS4A(1-48). As TEV protease recognizes E-X-X-Y-X-Q↓(G/S), and the first amino acid of the NS4A peptide is serine, a NS4A(1-48) peptide without any artificial overhang can be produced with this vector as well.

Third, we engineered a plasmid for expression of NS4A(1-48) as protein fusion with an amino terminal glutathion-S-transferase (GST) following the strategy described above for pGEV-NS4A(1-48). The insert coding for NS4A(1-48) was ligated into a dephosphorylated pGEX-4T-2 vector. The resulting construct was named pGEX-TEV-NS4A(1-48).

#### NS4A(1-48) plasmid construct with a dual fusion tag

Efficient separation of NS4A(1-48) from GB1 after TEV protease cleavage was impossible when utilizing pGEV-NS4A(1-48). In order to avoid this problem we extended the GB1-NS4A(1-48) fusion by an amino terminal GST affinity tag. For this purpose the GB1-NS4A(1-48) sequence was amplified with a 5′-primer containing an *Eco*RI site (7, table 1) and a commercially available T7-terminator primer (8, table 1). The restricted insert was cloned into the pGEX4T-2 vector using the *Eco*RI and *Xho*I sites. The obtained vector pGEX-GB1-NS4A(1-48) was used to produce the GST-GB1-NS4A fusion protein in *E. coli*.

Tail-to-tail mutagenesis was used to obtain the mutant NS4A peptide carrying L6E and M10E substitutions (pGEX-GB1-NS4A(1-48; L6E, M10E) [23] using kappa HiFi DNA polymerase (Kapa Biosystems Cambridge, MA, USA). The forward primer (11, table 1) carried the mutations while the reverse primer (12, table 1) annealed to the opposite strand, with their 5′-ends adjacent to each other. Subcloning into pGEX4T-2 was performed as described for the wild type peptide. The correct sequence of all constructs was verified using DNA sequencing (Seqlab, Göttingen, Germany).

### Expression of NS4A(1-48) fusion proteins

BL21 cells were transformed with pGEX-TEV-NS4A(1-48), pGEX-GB1-NS4A(1-48) or the mutant plasmid pGEX-GB1-NS4A(1-48; L6E, M10E). BL31(DE3) cells were used for pUbi-NS4A(1-48) and pGEV-NS4A(1-48), respectively. Recombinant protein was produced in LB medium supplemented with the appropriate antibiotics (ampicillin or kanamycin, 100 µg/ml). Uniformly [^15^N] or [^13^C, ^15^N] isotope-labeled NS4A(1-48) was expressed at 37°C in M9 medium containing [^13^C_6_] or [^12^C_6_] glucose and [^15^N] ammonium chloride as the sole carbon and nitrogen sources, respectively. Each 1 L expression medium was inoculated with an aliquot of a 50 ml overnight culture to an optical density of 0.1 at 600 nm (OD_600_). Gene expression was induced at an OD_600_ of 0.8 by addition of an ITPG stock to a final IPTG concentration of 1 mM. Cells were incubated under gentle agitation (150 rpm) and harvested 5 hours after induction by centrifugation (5000× g, 4°C, 30 min). Cell pellets were washed once in PBS buffer, spun down and stored at −20°C. Protein expression was verified using SDS-PAGE with Coomassie staining.

### Purification of GST-GB1-NS4A(1-48) fusion proteins and their proteolytic cleavage

Pellets of pGEX-GB1-NS4A(1-48) or pGEX-GB1-NS4A(1-48; L6E, M10E) transformed cells harvested from 1 L expression culture, respectively, were thawed and resuspended on ice in 25 ml lysis buffer (50 mM Tris-HCl pH 8.0, 150 mM NaCl, 1 mM DTT, 0.5 mM EDTA) supplemented with protease inhibitors (Complete mini, Roche, Penzberg, Germany). Cells were lysed using 4-5 cycles in a Microfluidizer M-100P (Microfluidics, Worcestershire, UK). The crude lysate was clarified by centrifugation (50000× g, 4°C, 30 min). Subsequent purification steps were performed at 22°C. The supernatant was applied onto a gravity flow column (column volume (CV) of 5 ml) packed with GSH sepharose 4B (GE Healthcare) and pre-equilibrated with lysis buffer. Unbound material was removed by washing with 10 CV of lysis buffer. When utilizing the mutant plasmid, on column cleavage of the GST-GB1-NS4A(1-48; L6E, M10E) fusion protein with 10 µM TEV protease (corresponding to a peptide to TEV ratio of about 100) was performed in standard buffer at 22°C overnight. The flow-through as well as the wash fractions (5 CVs) containing the free NS4A(1–48; L6E, M10E) peptide and TEV protease were pooled and concentrated to 5 ml using a Vivaspin 20 centrifugal concentrator (MWCO: 3 kDa, Sartorius, Göttingen, Germany). Separation of TEV protease from NS4A mutant peptide was accomplished with a HiLoad 16/60 Superdex 75 prep grade column (GE Healthcare) on an ÄKTA purifier system at 22°C with a flow rate of 1 ml/min. Interestingly, the GST-GB1-NS4A(1–48) wild type fusion protein could not reliably be digested under these standard conditions. Here, cell lysis had to be performed in lysis buffer supplemented with 0.5 M urea. GSH-binding and on column cleavage had to be done in the presence of 0.5 M urea and at a higher TEV concentration (50 µM, peptide to TEV ratio of about 10). Flow-through and wash was collected after TEV digestion and concentrated using Vivaspin 20 centrifugal concentrator. TEV-protease and urea was removed from the concentrated peptide solution using a Highload 16/60 Superdex 75 prep grade column, equilibrated in 50 mM Tris-HCl pH 8, 150 mM NaCl at a flow rate of 1 ml/min. NS4A containing fractions were pooled and concentrated. Cleavage, separation efficiency and final purity of the NS4A peptides were evaluated using SDS-PAGE analysis, and concentrations were calculated by measuring the absorption at 280 nm. NS4A(1–48) and the mutant peptide were kept at 4°C for short-term storage or rapidly frozen in liquid nitrogen prior to long-term storage at −80°C.

### TEV protease expression

TEV protease was produced using the plasmid pRK793 (Addgene). This plasmid codes for TEV fused to highly soluble maltose-binding protein that cleaves itself *in vivo* to generate a His-tagged TEV protease catalytic domain. Because of its S219V mutation this enzyme does not show the wild type typical auto-inactivation and allows the production of a stable and highly active enzyme. Expression and purification of the protease was performed as described [24].

### Mass spectrometric analysis

Identity of the NS4A peptides was confirmed using mass spectrometry. NS4A(1–48) peptide bands were excised from SDS polyacrylamide gels and subjected to tryptic digestion. Peptide fragment analysis was performed using an LC-MS/MS instrument (nanoUPLC-QTOF Premier, Waters Corp., Milford, MA, USA). These experiments were performed at the technology platform Integrated Functional Genomics of the Interdisciplinary Center for Clinical Research of the University of Münster, Germany.

### NMR spectroscopy

2D (^1^H-^15^N)-BEST-TROSY spectra [25] were recorded at 303.15 K on a Varian ^Unity^INOVA NMR spectrometer equipped with a cryogenic Z-axis PFG triple resonance probe operating at a proton frequency of 600 MHz. Data were processed with NMRPipe [26] and analyzed with CcpNmr analysis [27]. NMR samples contained 1 mM [^15^N] labeled NS5A(1–48) or NS4A(1–48; L6E, M10E), respectively, in 50 mM sodium phosphate buffer (pH 6.8) with 10% (v/v) deuterium oxide and 0.03% (w/v) NaN_3_.

## Results and Discussion

### Codon adaption of the NS4A(1–48) coding sequence

**Figure 1:**
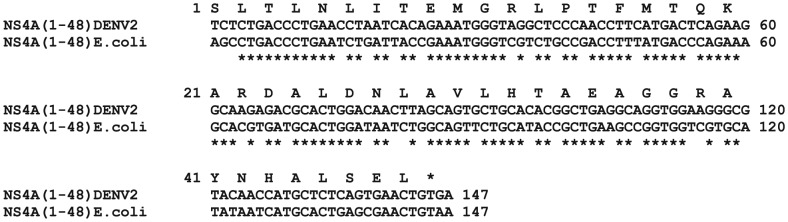
NS4A(1–48) coding sequence. ClustalW alignment [40] of the NS4A(1–48) coding sequence as found in the viral genome of DENV type 2 and following optimization for *E. coli* expression [21].

Only negligible amounts of NS4A peptide could be obtained when the original DENV type 2 cDNA sequence encoding the first 48 residues of NS4A, NS4A(1–48), was used in a pGEX expression vector. This may be explained by the fact that the viral DNA sequence for NS4A(1–48) is not optimal for expression in *E. coli* cells, due to different codon usage. Specifically several codons for leucine, isoleucine and arginine occurring in the viral gene sequence are “rare codons” in *E. coli* (Fig. 1). Rare codons are known to cause translational problems such as low protein expression and frame shifts [28]. Thus the codons were optimized for expression in *E. coli* cells and chemically synthesized oligonucleotides were used to create a synthetic NS4A(1–48) DNA cassette, which was then used to generate several NS4A(1–48) expression constructs.

### Expression yields of NS4A(1–48) strongly vary between different fusion tags

**Figure 2:**
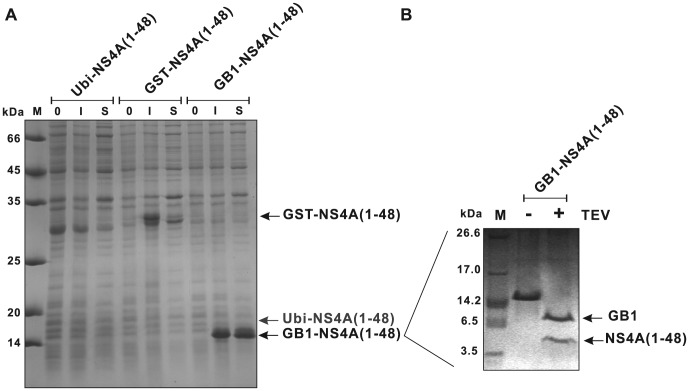
Comparative expression of NS4A(1–48) constructs containing single fusion tags. (A) SDS-PAGE analysis of the relative expression levels of NS4A fusions with ubiquitin (Ubi-NS4A(1–48)), glutathion-S-transferase (GST-NS4A(1–48)) and immunoglobulin-binding domain of streptococcal protein G (GB1-NS4A(1–48)). Aliquots of the expression cultures taken before (0) or 3 hours after IPTG induction (I) were applied. Aliquots of the supernatants after cell lysis (S) are shown as well. (B) TEV cleavage of the purified GB1-NS4A(1–48) fusion protein. Purified GB1-NS4A(1–48) fusion protein after size exclusion chromatography before (−) and after (+) TEV digestion together with a molecular weight marker (M; M3546, Sigma) were applied.

To identify the ideal conditions for NS4A(1–48) expression, the expression levels of codon-optimized NS4A(1–48) linked to three different kinds of fusion tags in *E. coli* cells were tested (Fig. 2A). *E. coli* BL21 harboring pGEX-TEV-NS4A(1–48) and BL21(DE3) cells harboring pUbi-NS4A(1-48) or pGEV-NS4A(1–48) were induced with IPTG for the indicated time, a band corresponding to the full-length NS4A(1–48) fusion protein was detected in the Coommassie-stained gel for the GST and the GB1 fusions. A full-length NS4A(1–48) fusion protein could not be detected for Ubi-NS4A(1–48) using Commassie staining. However, western blot analysis confirmed its expression (data not shown). Highest expression levels of NS4A(1–48) were detected with the GB1 fusion tag. As all the plasmids contained the same optimized NS4A sequence the GB1 domain seems to account for these high expression levels presumably by stabilizing the NS4A(1–48) peptide. In addition, only the GB1-NS4A(1–48) fusion protein could be efficiently extracted to the soluble fraction after cell lysis without adding detergents. These findings are in line with previous observations [18], [29]. The average yields for the three expression constructs are summarized in the upper part of table 2. The highest amount of target peptide was produced with pGEV-NS4A(1–48). Subsequently, the GB1-NS4A(1–48) fusion was purified as described [18], however, TEV protease was used instead of Factor Xa. The optimum recognition site for TEV is Glu-Asn-Leu-Tyr-Phe-Gln-(Gly/Ser) (ENLYFQ(G/S)) and cleavage occurs between the Gln and Gly/Ser residues [30]. DENV NS4A starts with serine and thus TEV cleavage allows a very specific and efficient removal of the fusion tag without any artificial overhang. Briefly, the GB1 fusion protein was extracted from the cell lysate by a simple heating step followed by size exclusion chromatography yielding approximately 90% pure fusion protein. TEV cleavage of the GB1 fusion protein was complete after about 16 h when using a fusion protein to protease ratio of about 100 (Fig. 2B). However, we were not able to efficiently separate the cleaved NS4A(1–48) peptide from the GB1 domain. Separation by size exclusion chromatography was hampered by similar elution behavior of both molecules, which have similar molecular weights (5223.7 Da for NS4A(1–48) and 7585.3 Da for GB1). Thus IgG sepharose affinity chromatography was used following an established protocol [22]. However, the GB1 domain could not be completely removed even when IgG sepharose was applied in large excess. This suggests that the heating step might have caused misfolding of a substantial fraction (up to 20%) of GB1 causing the observed leakage during affinity chromatography. Subjecting the digest reaction to diverse reverse phase chromatography resins did not yield satisfying results either. The target NS4A(1–48) peptide showed a strong tendency to remain on the column even at high acetonitrile concentrations, presumably due to its amphipathic nature (data not shown). Consequently, the highest purity that could be achieved for untagged NS4A(1–48) was about 80% with an average recovery of 60% (see Table 2).

**Table 2:** Average yields and purities of the studied NS4A(1–48) fusion proteins and of the resulting NS4A(1–48) target peptides obtained from 1 L of culture.

Fusion Tag	Yield											
	NS4A(1–48) Fusion Protein						NS4A(1–48) Peptide					
	after first purification step						after proteolytic cleavage and tag separation					
	*Wild Type*			*Mutant*			*Wild Type*			*Mutant*		
	amount (mg)	purity (%)	NS4A(1–48) content (mg)	amount (mg)	purity (%)	NS4A(1–48) content (mg)	amount (mg)	recovery (%)	purity (%)	amount (mg)	recovery (%)	purity (%)
*GB1*	15	90	6.1	20	90	8.1	3	50	<80	5	62	<80
*GST-GB1*	25	60	3.3	30	75	4	3	91	∼98	4	∼100	∼98
GST-GB1*	35	60	4.7	40	75	5.3	4	85	∼98	5	94	∼98

Due to the different sizes of the fusion tags (Ubi: 11.3 kDa, GST: 26.4 kDa, GB1: 7.6 kDa, GST-GB1: 34 kDa) the mass fractions of the target peptide differ significantly between the constructs (Ubi: 31.5%, GST: 16%, GB1: 40.6%, GST-GB1: 13.3%). Since Ubi and GST fusion constructs resulted in very low yields (>1 mg/l), data for these constructs have not been included. The values for GB1 and GST-GB1 have been used to calculate the theoretical NS4A(1–48) content after the first purification step for each of the constructs shown. Despite an unfavorable target peptide to fusion tag ratio highest recovery and purity values were obtained with the dual GST-GB1-NS4A(1–48) construct after proteolytic cleavage and tag removal. LB medium was used if not stated otherwise. An asterisk (*) indicates isotope ([^13^C, ^15^N] or [^15^N]) labeled minimal growth medium. Note that the overall peptide yields were higher in minimal medium compared to those in rich LB medium. Amount and purity of the fusion protein tagged peptides as well as the purity of the free peptides has been estimated from SDS PAGE analysis. Final free peptide yields were additionally calculated by measuring the concentration of the peptides at 280 nm using an extinction coefficient of 1490 M^−1^ cm^−1^ in water.

### Additional tagging of GB1-NS4A with GST allows a simplified separation of NS4A(1–48) from the fusion tag

**Figure 3:**
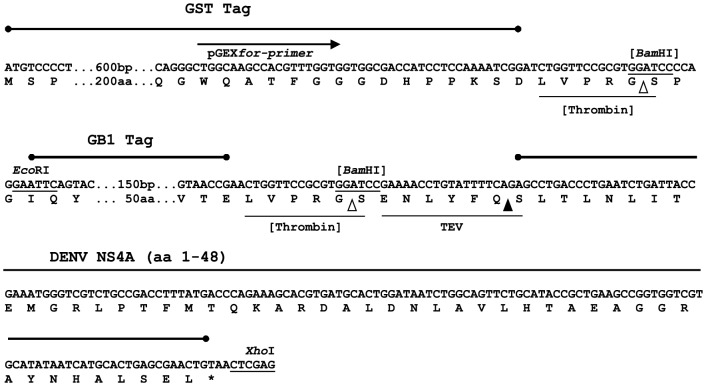
Expression/cloning region of pGEX-GB1-NS4A(1–48). The GB1-NS4A(1–48) sequence was inserted into pGEX-4T-2 between the *Eco*RI and *Xho*I sites. Protease recognition motifs are underlined while the cleavage sites are marked by triangles. The two thrombin sites, which originate from the vector backbone of pGEX and pGEV, respectively, are shown in brackets and were not used in our protocol. Note that TEV digestion produces a native NS4A peptide.

**Figure 4:**
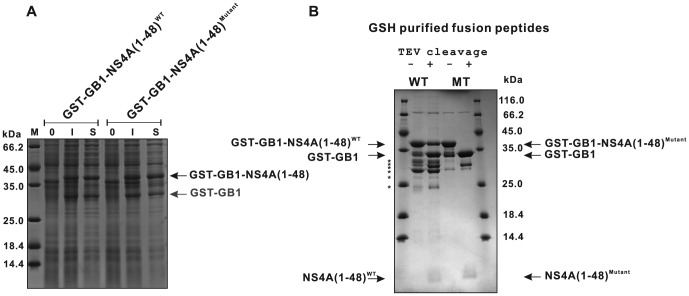
Expression of NS4A(1–48) wild type and mutant peptides using a dual GST-GB1 tag. (A) Relative expression levels of GST-GB1-NS4A(1–48) and the GST-GB1-NS4A(1–48; L6E, M10E) mutant were analyzed by SDS-PAGE using aliquots of the expression cultures. Shown are samples obtained from culture at 0 (0) or 3 hours (I) following IPTG induction or the supernatant after cell lysis (S). (B) TEV digest of GST-GB1-NS4A(1–48) wild type and mutant protein fusions. Aliquots of GSH-purified supernatants of wild type and mutant fusion proteins before and after TEV cleavage are shown. Note that besides the GST-GB1-NS4A(1–48) full-length product also shorter fragments, likely GST-GB1 and other truncation fragments, marked by asterisks were produced, which are present in the GSH-purified samples already prior to TEV cleavage. Because staining of free NS4A(1–48) peptides is very faint under the conditions used, the progress of the TEV digest is monitored by observing the decrease of the band for the dual tagged GST-GB1-NS4A(1-48) fusion protein in parallel with an increase of the band for the free GST-GB1 dual tag. A densitometric analysis of the respective bands revealed a cleavage efficiency of approximately 50% for the wild type peptide.

Due to the difficulties in separating NS4A(1–48) from GB1 after TEV protease cleavage as described above, we decided to add an additional amino terminal GST tag to the protein (Fig. 3). The relative expression levels achieved with this construct and the respective mutant construct were evaluated using SDS-PAGE (Fig. 4A). The data demonstrate proper and stable expression of NS4A(1–48) fusion proteins. The GST-GB1 fusions could be easily purified using GSH sepharose. The average yields of the GST-GB1-NS4A peptide fusions are given in Table 2. TEV cleavage under standard conditions could efficiently remove the dual tag from the NS4A(1–48; L6E,M10E) mutant peptide. However, the GST-GB1-NS4A(1–48) wild type construct showed unexpectedly low cleavage efficiency (Fig. 4B).

### Addition of urea improves the TEV cleavage of the wild type NS4A(1–48) peptide

**Figure 5:**
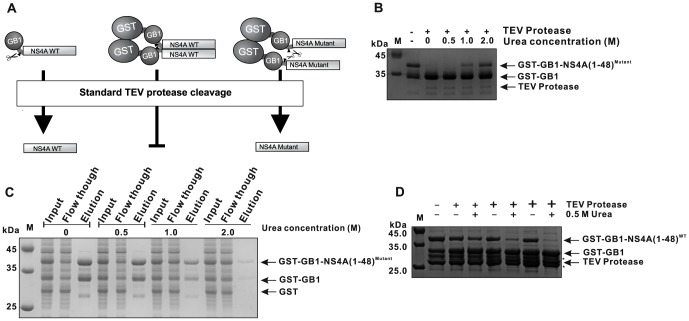
Optimization of TEV protease cleavage conditions. (A) A schematic model explaining the resistance of the GST-GB1 dual tagged wild type NS4A(1–48) peptide to TEV cleavage. Scissors illustrate the protease while the triangles represent the position of the cleavage sites. GST induced oligomerization of wild type NS4A(1 48) may block the protease cleavage site. (B) Analysis of the urea tolerance of TEV protease activity. GST-GB1 tag removal from NS4A(1 48; L6E, M10E) in the presence of different concentrations of urea (M). Fusion peptides were incubated with TEV protease at 20°C for 16 h at a fusion peptide to protease molar ratio of approximately 100. (C) Binding properties of GST-GB1-NS4A(1–48) to GSH sepharose in the presence of urea. Equal amounts of the fusion peptide were subjected to a mini-scale GSH-affinity chromatography in the presence of different concentrations of urea (M). Input, flow-through and elution fractions were analyzed by 15% SDS-PAGE. (D) TEV cleavage efficiency of wild type NS4A(1–48) peptide in the presence of urea. GST-GB1 tag removal from NS4A(1–48) in the presence of different concentrations of urea given in Molars. Fusion peptides were incubated in the absence (−) or presence (+) of TEV protease at 20°C for 16 h. The different “+” font sizes indicate the increasing amounts of TEV protease with fusion peptide to protease molar ratios of approximately 100, 50 and 10. The progress of the TEV digest is monitored by observing the decrease of the band of the dual tagged GST-GB1-NS4A(1–48) fusion protein and a parallel increase of the free GST-GB1 dual tag band (B, D).

GST is known to form dimers in solution [31]–[33]. Examples of GST-induced oligomerization of GST fusion proteins can be found in the literature [34]–[36]. Such a GST-induced oligomerization might also explain the observed resistance of a substantial fraction of wild type GST-GB1-NS4A(1–48) to TEV cleavage under standard conditions (Fig. 4B). Previous results from our laboratory indicate that the amino terminal AH of NS4A plays a role in the homo-oligomerization of NS4A [6]. We speculated that the purified GST-GB1-NS4A(1–48) peptides might self-associate and thus block the TEV recognition site (see Fig. 5A for explanation). The NS4A mutant peptide, which is expected to show a reduced self-association, was easily cleaved supporting the above notion. We optimized the TEV digestion reaction conditions with the aim to decrease the NS4A peptide self-association without reducing the proteolytic activity of TEV. Denaturing or chaotropic reagents like urea are typically used to break up such aggregates. TEV activity studies by Sun et al. suggested that a recombinant TEV protease can retain most of its activity at relatively high concentrations of denaturants such as 2 M urea [37]. In contrast Waugh *et al.* reported a lower urea tolerance of 0.5 M for TEV [38]. Therefore, we first tested the GST-GB1-NS4A(1–48; L6E, M10E) mutant construct, which easily can be digested without additives, to assay the urea tolerance of TEV protease under our experimental conditions. At low urea concentrations (up to 0.5 M), no loss of TEV activity was observed (Fig. 5B). Higher urea concentrations (1–2 M) resulted in a significant activity loss of more than 50%, as described by Waugh [38]. Our results indicate that the NS4A peptide aggregation is perhaps a cooperative process. Therefore, addition of chaotropic molecules early on in the purification process might further improve the final yield of target protein. In order to add urea already to the lysis buffer further optimization was required. It was important to ensure that GST binding to GSH sepharose will not be affected by the added urea. An earlier study indicated that GST binds to GSH sepharose in presence of chaotropic reagents like 2–3 M guanidine hydrochloride or urea [39]. We independently assessed the binding properties of GST-GB1-NS4A(1–48) to GSH sepharose in the presence of increasing urea concentrations (Fig. 5C). Addition of 0.5 M of urea did not alter the binding behavior of GST to GSH sepharose. However, higher urea concentrations resulted in a drastic shift of the GST-GB1-NS4A(1–48) peak from the elution to the flow-through fractions. Next we assayed the TEV cleavage efficiency for the double tagged wild type NS4A(1–48) fusion construct in the presence of increasing urea concentrations. Conveniently, 0.5 M urea was sufficient to initiate the removal of GST-GB1 tag (figure 5D). Yet, TEV protease amounts had to be increased at least 5-fold compared to the cleavage reaction of the mutant peptide. Nevertheless, under these conditions, nearly complete removal of GST-GB1 from the wild type NS4A(1–48) peptide was achieved.

### The GST-GB1 fusion allows a nearly identical purification protocol for both wild type and mutant NS4A peptides

**Figure 6:**
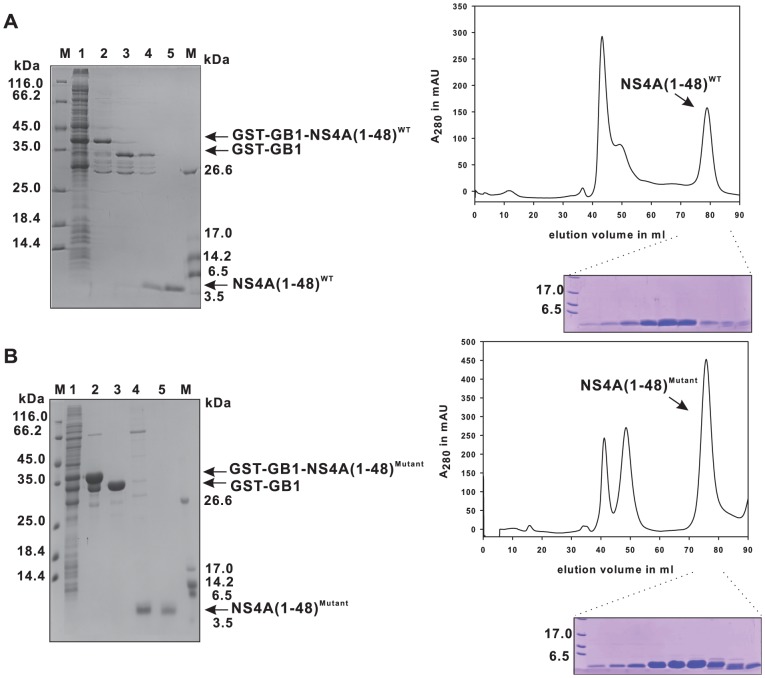
Purification of recombinant NS4A(1–48) wild type and mutant peptides. The 15% SDS-PAGE gel containing samples from various steps in the purification procedure is shown on the left. The wild type peptide is shown in (A) while the mutant is shown in (B). The supernatant after cell lysis is shown in lane 1. The lysate containing the peptide was loaded on a GSH sepharose column (lane 2), and cleaved by TEV protease on the column, the cleaved protein is shown in lane 3. The GSTfusion tag remained bound to the column, while the NS4A(1–48) peptide was collected from the flow-through (lane 4). The peptide was further purified by size exclusion chromatography (lane 5). The mutant peptide was purified using the same strategy (B). The respective size exclusion chromatography profiles (HiLoad 16/60 Superdex 75 prep grade) of the flow-through fraction from the TEV protease on column cleavage step - mainly containing TEV protease and NS4A(1–48; L6E, M10E) or NS4A(1–48) peptides - are shown on the right with the matching SDS-PAGE analysis of the NS4A containing fractions.

Production of diverse mutant forms of NS4A(1–48) besides the wild type is necessary for an in-depth structure-function-analysis. Our aim was, to establish a general purification procedure applicable to various NS4A(1–48) mutant peptides. Therefore, purification steps that are sensitive to changes in peptide charge or hydrophobicity, as it would be the case when applying ion exchange or reverse phase chromatography, were avoided. The feasibility of our simple purification procedure, which is based exclusively on GSH-affinity and size exclusion chromatography, is demonstrated for the wild type peptide and for the NS4A(1–48; L6E, M10E) mutant as an example. This NS4A(1–48) mutant carries glutamate substitutions at positions 6 and 10 instead of leucine and methionine, respectively, resulting in a shift of the isoelectric point from 6 to 5 and a considerably decreased hydrophobicity of the peptide. The purification progress for the wild type and the mutant NS4A(1–48) peptides is summarized in figure 6. In both cases highly purified peptides could be obtained after size exclusion chromatography. The identity of the peptides was confirmed by in-gel tryptic digestion of the electrophoretically separated protein band and mass spectrometric peptide mapping. As summarized in table 2 we could obtain up to 3 mg untagged wild type and up to 4 mg mutant peptide of very high purity (approx. 98%) from 1 liter culture in rich medium. These values indicate an almost complete TEV cleavage as well as an efficient separation of the peptides from the dual tag, which was not possible with the GB1 single tag system. Thus, our dual tag strategy provides an efficient way for producing NS4A(1–48) in *E.coli*.

### Peptide labeling with stable isotope and initial nuclear magnetic resonance spectra

**Figure 7:**
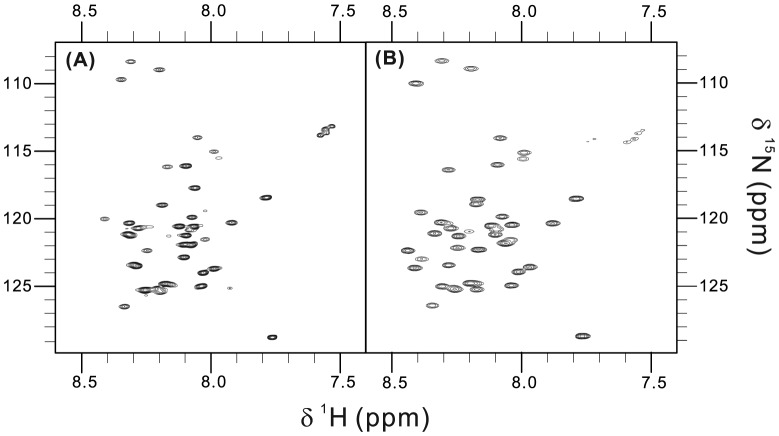
Recorded 2D (^1^H, ^15^N)-BEST-TROSY spectra of the purified NS4A peptides. Spectrum of 0.5[^15^N]-NS4A(1–48) wild type (A) and of 1 mM mutant (L6E, M10E) peptide (B) in 50 mM sodium phosphate buffer, pH 6.8. Data were recorded at 30°C.

Peptides labeled with the stable isotopes ^15^N and ^13^C can be studied in great detail by nuclear magnetic resonance (NMR) spectroscopy. Solution NMR is the method of choice for the structural investigation of conformationally flexible peptides. Using the double tagged GST-GB1-NS4A(1–48) fusion protein we could easily produce even higher amounts of labeled NS4A(1–48) by growing the *E. coli* cells in minimal medium supplemented with [^15^N] ammonium chloride with either [^12^C_6_] or [^13^C_6_] glucose as sole nitrogen and carbon sources (Table 2). Production of uniformly ^15^N-labeled NSA4(1–48) peptides in minimal medium resulted in very similar peptide yields and purity as in case of doubly labeled peptide. 2D (^1^H-^15^N)-BEST-TROSY spectra of NS4A wild type and mutant peptides in aqueous buffer are shown in figure 7. The position and low spectral dispersion of the observed amide ^1^H-^15^N cross signals indicate unstructured peptides. This is in good agreement with our earlier circular dichroism (CD) data on these two peptides in aqueous buffer [6]. These results indicate that the amount of highly pure isotope-labeled NS4A peptides produced using the described double tag strategy is sufficient for future multidimensional NMR experiments. These experiments are required for peptide resonance assignment and for gathering information regarding the structure of the peptides and their dynamics in presence of membranes or membrane mimetic model systems.

## Conclusion

Our GST-GB1 fusion approach represents a valuable tool for preparing milligram amounts of unlabeled or isotope labeled NS4A(1–48) peptides. This is an important prerequisite for a detailed analysis of the structure-function relationship of the amino terminal region of NS4A with the aim to elucidate the mechanism how this region interacts with membranes. The results may lead to new antiviral strategies to fight DENV. In general the presented production procedure may also aid the characterization of other aggregation prone proteins that are frequently coded in viral genomes.
